# Acoustery System for Differential Diagnosing of Coronavirus COVID-19 Disease

**DOI:** 10.1109/OJEMB.2021.3127078

**Published:** 2021-11-10

**Authors:** A. Yu Mitrofanova, D. Mikhaylov, I. Shaznaev, V. Chumanskaia, V. Saveliev

**Affiliations:** Bauman Moscow State Technical University 65024 Moscow 105005 Russia; Lebedev Physical InstituteRussian Academy of Sciences 54744 Moscow 119991 Russia; Shanghai Jiau Tong University 12474 Shanghai 200240 China; Immanuel Kant Baltic Federal University 64920 Kaliningrad 236041 Russia; Huazhong University of Science and Technology 12443 Wuhan 430074 Hubei China

**Keywords:** Attention mechanism, convolutional neural network, COVID-19, preliminary diagnosis, recurrent neural network

## Abstract

*Goal:* Because of the outbreak of coronavirus infection, healthcare systems are faced with the lack of medical professionals. We present a system for the differential diagnosis of coronavirus disease, based on deep learning techniques, which can be implemented in clinics. *Methods:* A recurrent network with a convolutional neural network as an encoder and an attention mechanism is used. A database of about 3000 records of coughing was collected. The data was collected through the Acoustery mobile application in hospitals in Russia, Belarus, and Kazakhstan from April 2020 to October 2020. *Results:* The model classification accuracy reaches 85%. Values of precision and recall metrics are 78.5% and 73%. *Conclusions:* We reached satisfactory results in solving the problem. The proposed model is already being tested by doctors to understand the ways of improvement. Other architectures should be considered that use a larger training sample and all available patient information.

## I. Introduction

The COVID-19 pandemic has resulted in significant challenges for society. According to WHO (World Health Organisation) statistics there have been 236,599,025 confirmed cases of COVID-19, including 4,831,486 deaths all around the world on 8 October 2021. It is impossible not to appreciate the work of doctors who are faced with a huge number of patients. However, the COVID-19 (Coronavirus Disease 2019) pandemic has exposed some health problems, in particular the lack of medical professionals. Today, it is worth thinking about supplying hospitals with special software that can help a doctor to decide if there is a need for an urgent polymerase chain reaction (PCR) test and thus will reduce the burden on the laboratories and increase their efficiency. With the growing popularity of machine learning and deep learning methods, it is obvious to refer to this area to find a solution.

One can use audio records to diagnose some disease states as in some pre-pandemic work [1]. Their main idea is based on the processing of audio signals from the human body: coughing, breathing, chest sounds. In addition to processing the sounds of the human body, chest X-ray [2], [3] and CT (computed tomography) images [4], [5] are also used to diagnose COVID-19 using deep learning methods.

According to a study by Brown *et al.* at the University of Cambridge [6], simple binary classifiers using logistic regression, gradient boosting, and support vector machines give a precision of up to 82% for the task of classifying audio records with cough on COVID and Non-COVID classes. These methods use MFCC, roll-off frequency, spectral centroid, and other features as an input. In addition, the authors also solved the problem of binary classification into the categories of COVID and asthmatic cough, which led to 64% accuracy. Data collection for this project is carried out using a mobile and web applications.

Another project named *Coswara* [7] provides web-based recordings of coughing, breathing and speech in the public domain on the GitHub platform. A set of different temporal and spectral acoustic features were extracted from the audio files. Some of them are MFCC (Mel Frequency Cepstral Coefficients), spectral roll-off and spectral flatness. A random forest classifier was trained to classify every record segment into one of the nine sound categories. The accuracy of the classification using the random forest method on test data reached 66.74%.

In the work of Imran *et al.* [8], the *AI4COVID-19* classifier is presented as three branches and a mediator, similar to the independent opinions of several doctors. In the proposed implementation, a positive or negative result of COVID-19 is set only when the decisions of the three branches coincide, which reduces the probability of an error to $6.147 \cdot {10^{ - 4}}$. The classifiers use convolutional networks and support vector machines. Authors use two multi class classifiers. One of them utilizes Mel spectrograms as an input and the other uses MFCC (Mel Frequency Cepstral Coefficients) and PCA (Principal Component Analysis) based feature extraction. The third classifier is the deep transfer learning-based binary classifier, which utilizes Mel spectrograms as an input. There is no information about accuracy of the whole architecture, but Table III contains different metrics for separate classifiers.

Through a number of medical studies [9]–[11], significant differences have been identified between the cough of a healthy and a sick person. They are manifested in such spectral and temporal parameters as the frequency of the maximum sound energy, the total duration of the cough, the duration of each phase, etc. The works [6], [8], [12] use preliminary data processing aimed at extracting the frequency parameters of cough recording. At the same time, convolutional and LSTM classifiers are used in [12] to detect cough episodes, which demonstrate high detection accuracy.

The *Acoustery* project, which will be discussed in this work, was created to recognize patients with COVID-19. We assume that even without the stage of extracting the frequency features of the recording, it is possible to obtain an equally high-quality classification for COVID-19 infection by training convolutional and recurrent networks to diagnose the disease by the “appearance” of the spectrograms. In the future, the classifier will not only make it possible to successfully recognize people infected with the coronavirus but will also serve as an assistant for doctors in making diagnoses related to other respiratory diseases. However, a model has now been implemented using convolutional and recurrent networks, which reaches an accuracy of 85%. Data collection plays an important role in the project, namely, records of coughing, breathing and speech. At the moment, the database contains of about 3000 records of coughing and about 6000 more records of breathing and speech. Data is collected through the created mobile application with the help of medical professionals, in agreement with the ethics committees of hospitals. The collection of records of the cough of sick and healthy patients using the application is carried out under the noise conditions. Specialists in the field of machine learning and medicine process the received data.

## II. Materials and Methods

### A. Data Description

**FIGURE 1.**
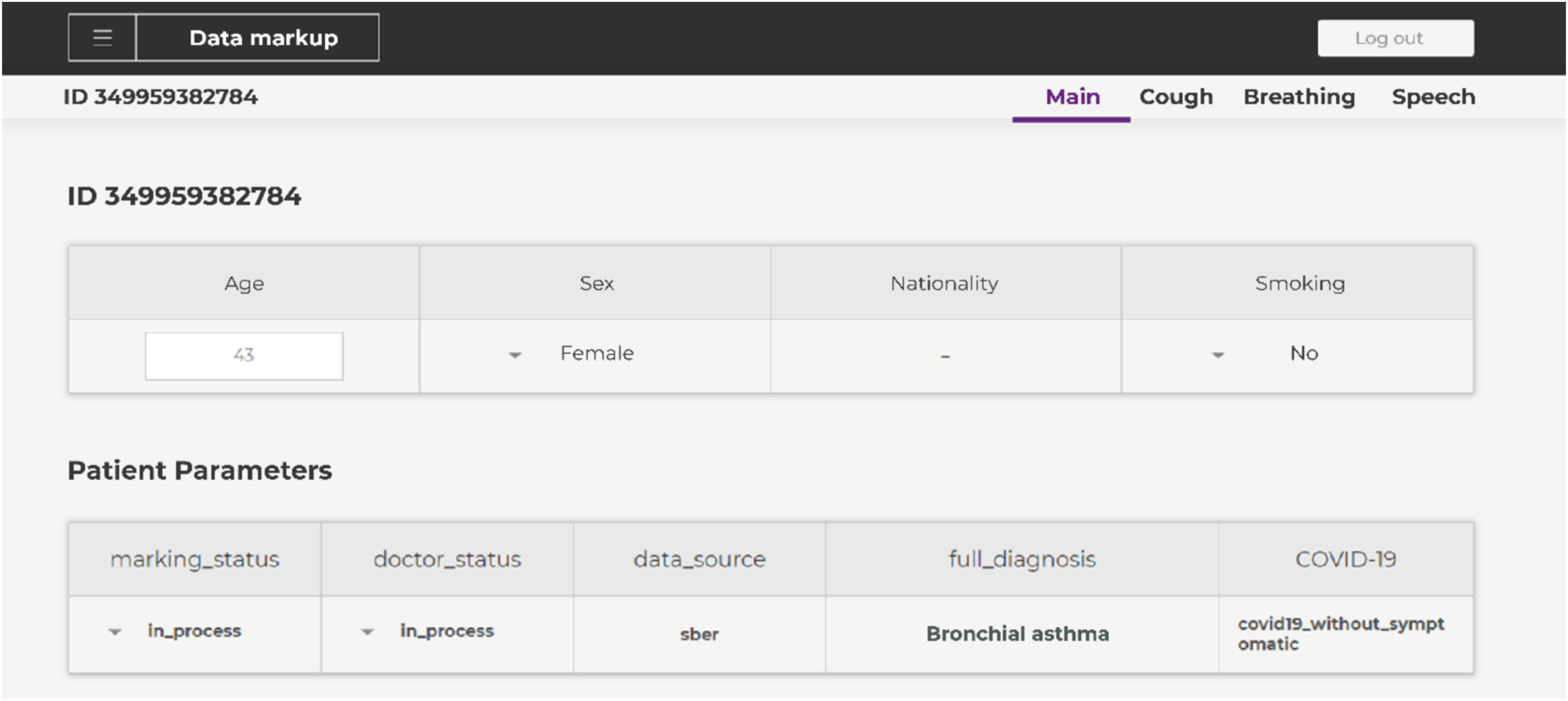
An example of completing basic information about a patient.

All methods of obtaining data, namely, a mobile application used in hospitals, available for Android and iOS, and publicly available recordings provide not only recordings of coughing, breathing and speech, but also other general data like gender, age, height, weight, etc. The presence of such information will allow it to be taken into account when making a diagnosis by the program in the future. In addition to general information, we also clarify some medical data, for example, the presence of chronic diseases and the fact of infection with COVID-19. An example of filling out basic information about a patient is shown in Fig. 1. In future work, it is necessary to expand the list of recorded symptoms by those that do not relate to breathing.

**FIGURE 2.**
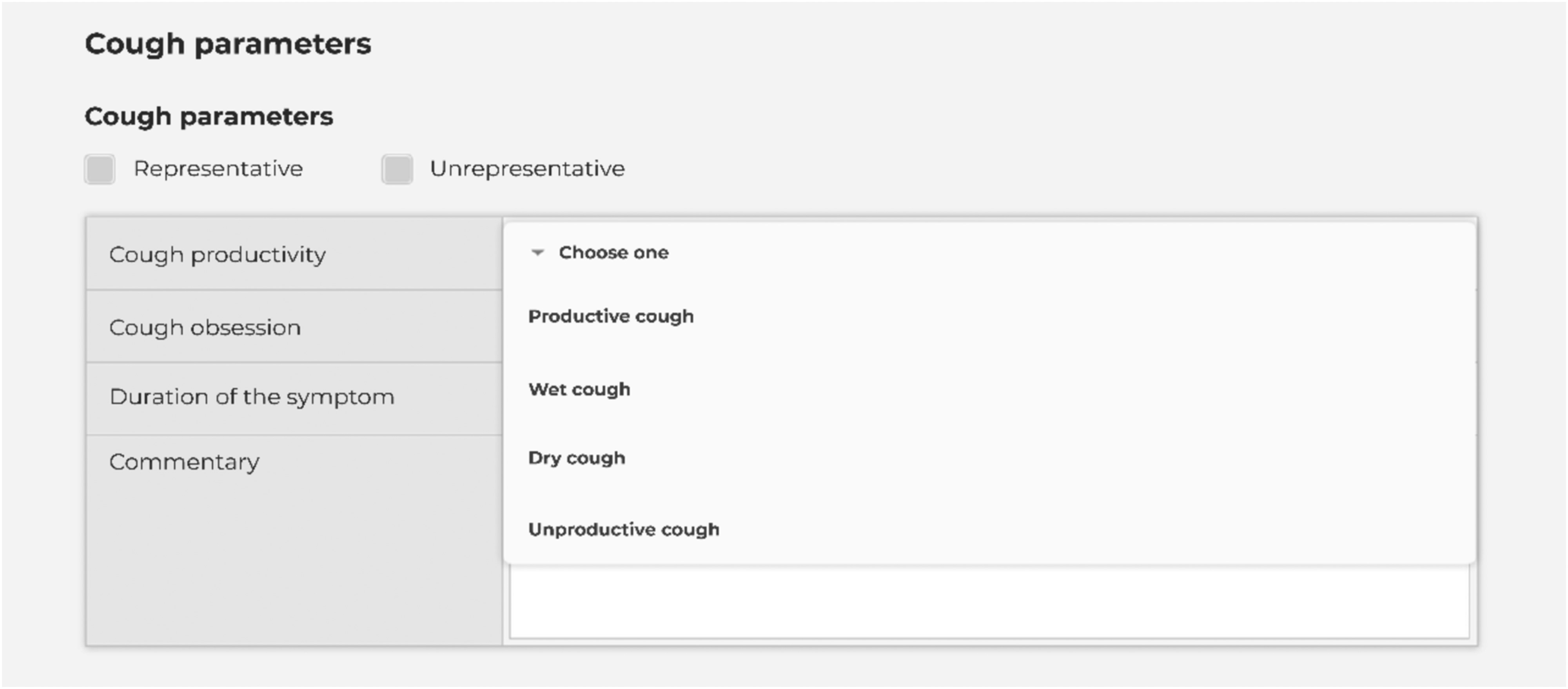
Form for doctors marking up records.

Because not only IT specialists, but also doctors are involved in the data labeling, for each record we have the characteristics of cough, inhalation, and exhalation, as well as preliminary diagnoses made by doctors based on the records. When marking up the data, the productivity and obsession of coughing are characterized, as well as breathing characteristics such as difficulty and duration of inhalation and exhalation. This information can be considered in the future when training the model to classify other respiratory diseases. Figs. 2, 4 illustrate the data labeling process. The labeling shown in Fig. 2 is a prime example of the involvement of medical professionals in processing records. In this case, they fill in the information that they deal with in medical practice, and which is the basis for the classification of the patient's illness.

**FIGURE 3.**
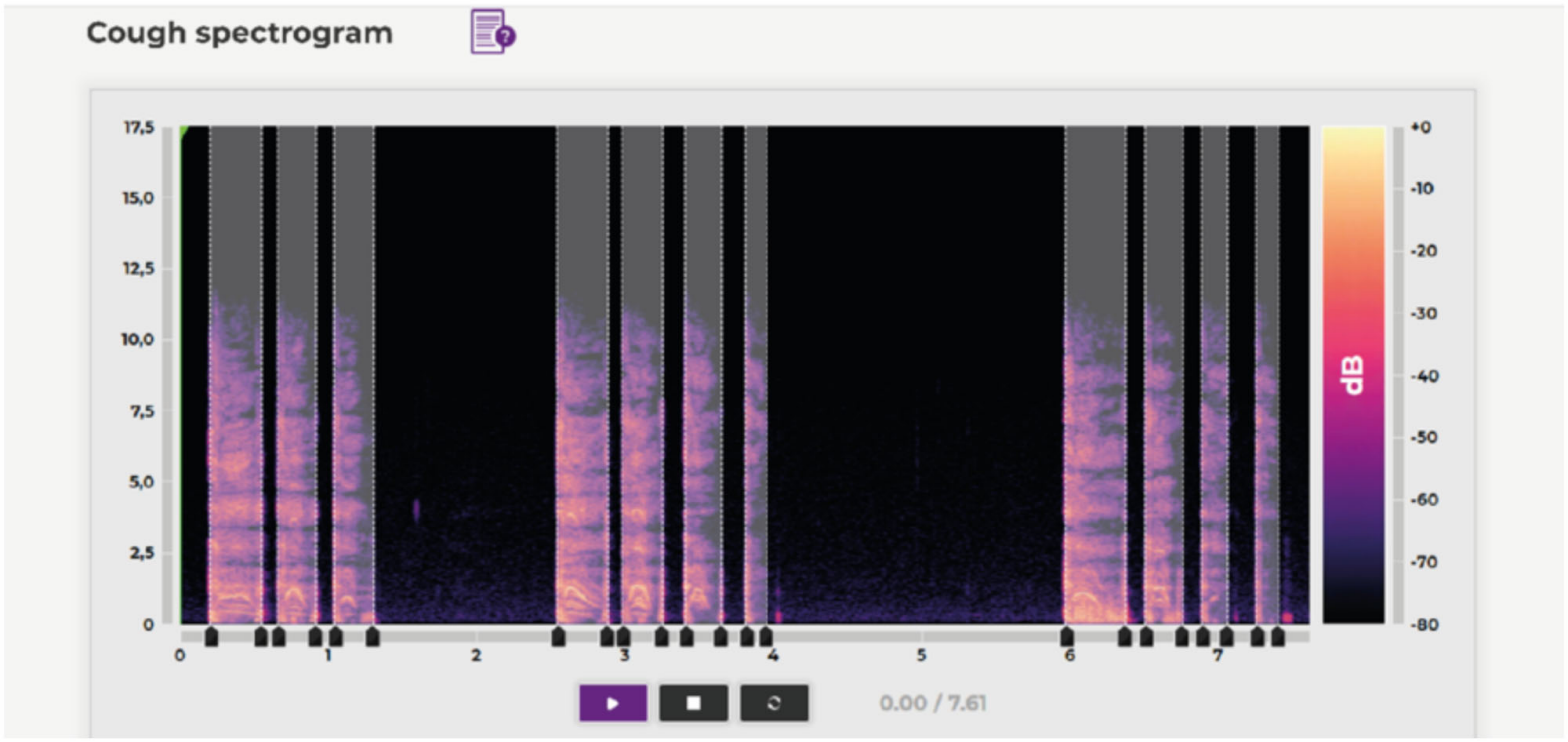
Marking cough impulses.

**FIGURE 4.**
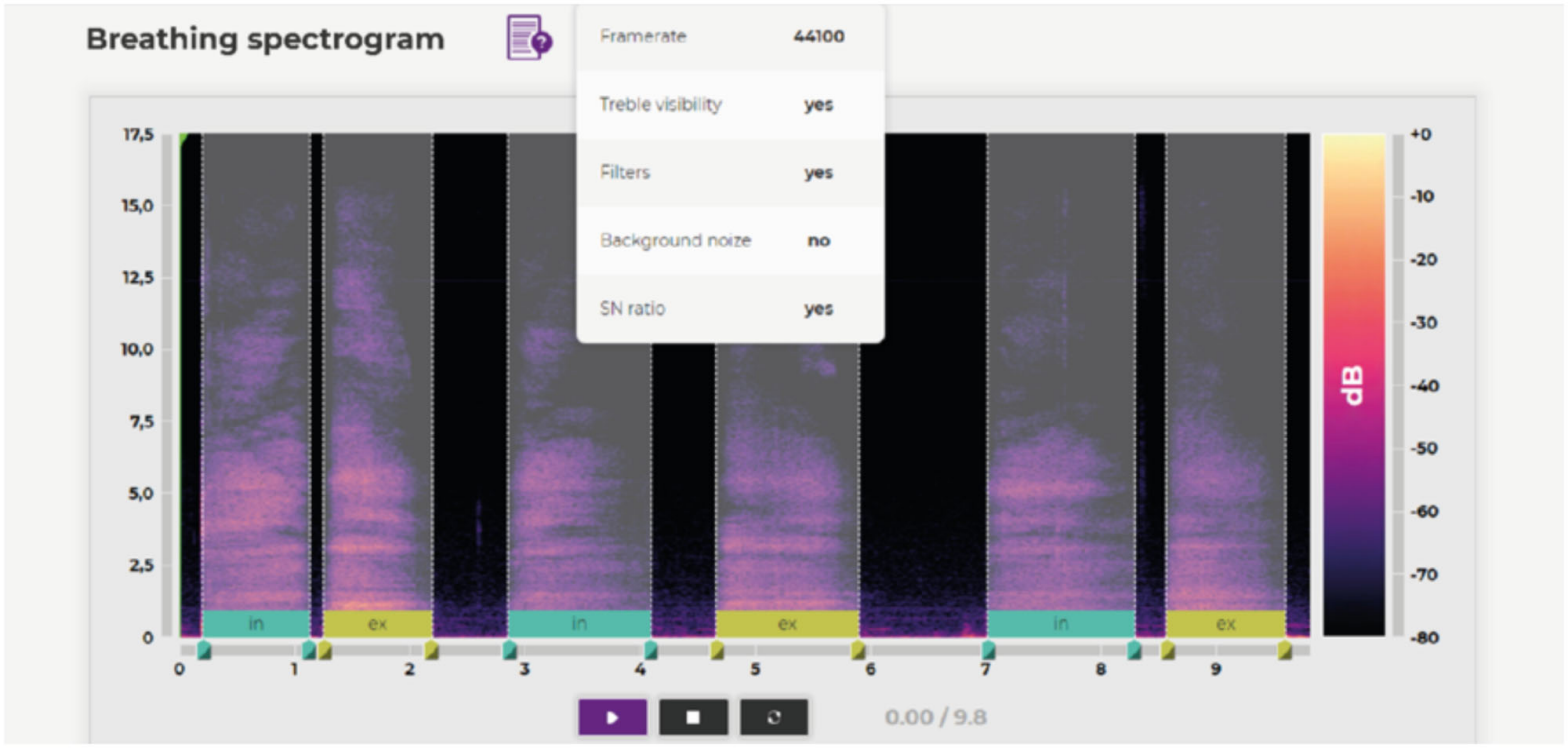
Marking inhales and exhales.

Information received from users is confidential since the system uses anonymized data. Registration does not require personal information (mobile phone or email). At any time, at the request of the patient, the doctor can delete the archive with the personal information of this user, which will lead to the complete removal of any of the patient's data from the *Acoustery* storage systems.

The problem for training the model is the significant predominance of records from people not infected with COVID-19. However, it should be noted that other projects have also faced this question. A similar distribution is also observed for other respiratory diseases. Therefore, for better training of the model, the observed ratio should be artificially changed. In contrast to the fact that the collected database contains more records of healthy people than infected, the number of records of coughing, speech and breathing is almost the same since the fact that each patient was asked to take turns to make records of breathing, speech and cough. Thus, the distribution between the recordings of breathing, coughing and speech is approximately the same for both COVID patients and those who are not infected with this virus. So far, we have focused on coughing as the most prominent symptom of respiratory disease and have decided to process those 300 records obtained using the app.

These records are the separate records of in total 300 patients, which are healthy and infected patients. The recording frequency corresponds to 44100 Hz. The audio format is wav. While working with the patient, the instructed doctors prepared the recording apparatus and asked them to cough. According to the doctors themselves, a considerable part of the records (which were not included in either the training or the test sample) of the cough of healthy patients turned out to be simulated. Forced cough is also more common in healthy patients, while passive cough is more common in sick patients. After receiving the recording, they were marked by medical specialists. They decided on the possibility of using a specific recording to diagnose a disease by the sound of a cough. Depending on this decision, the record fell or did not fall into the training set. IT specialists, on the other hand, standardized the recordings, which included removing noise and converting them to the same frequency range.

### B. Model Architecture

**FIGURE 5.**
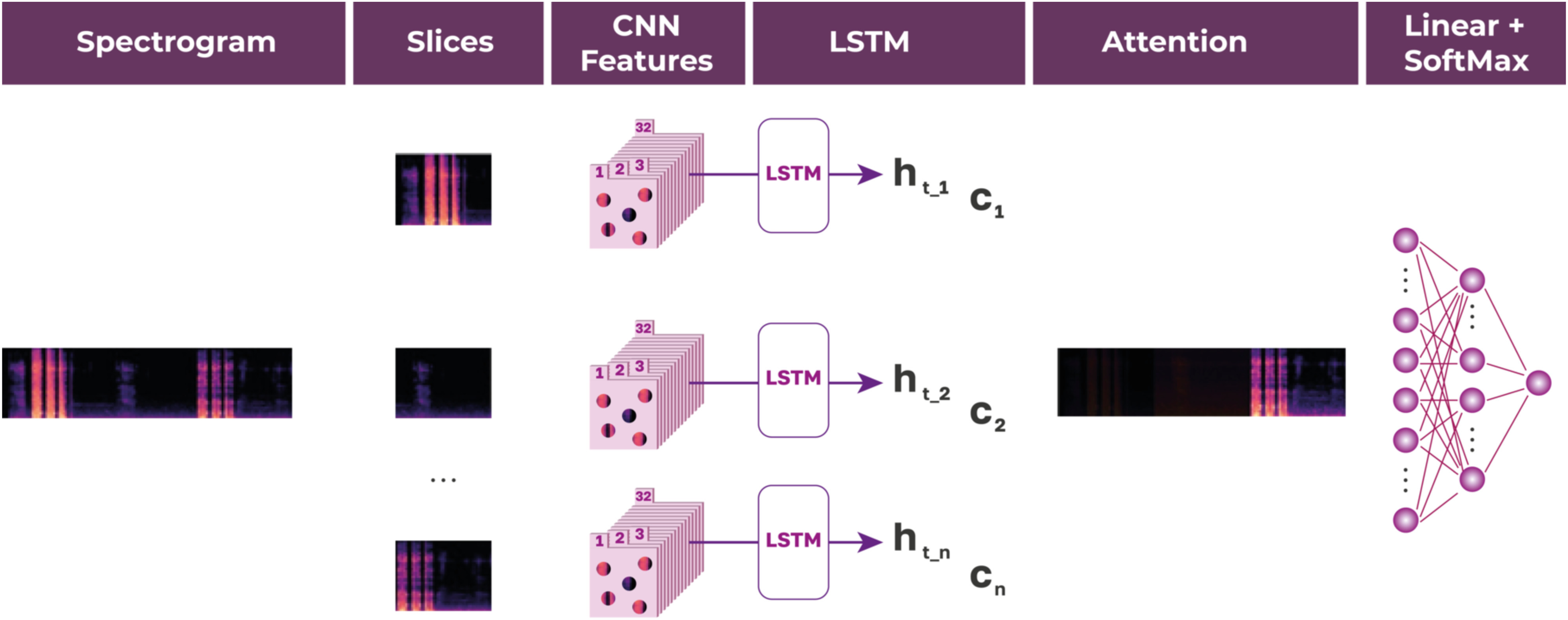
Classification model.

The model for recognizing patients with COVID-19 is illustrated in Fig. 5. It is a recurrent neural network with a CNN encoder and attention mechanism and linear layers following it. The encoder processes the cough spectrograms received as input data and consists of four blocks, including the convolution operation with a $3 \times 3$ kernel, an activation layer with the LeakyReLu function, a dropout method with the probability of excluding a neuron $p = 0.7$ to prevent overfitting, and batch normalization.

The features that the encoder extracted are then processed in the LSTM layer with the dimensions of the $k = 512$ internal gates. Note that the recurrent network does not work with the entire record, but with a fixed-size window corresponding to approximately 300 milliseconds of recording. The output of the LSTM layer goes through the attention mechanism, which is implemented using a linear layer, a hyperbolic tangent as its activation function and the scalar product of the desired weights and values of the activation function. The task of the attention mechanism is to identify information that is important for diagnosing a disease.

The found vector $c$ passes through a linear layer with the ReLu activation function, which halves its size. The probability of excluding a neuron in the dropout layer remains equal $p = 0.7$. On the next linear layer, we get one output value, which, after passing through the sigmoid, takes on the meaning of the probability of a person having COVID-19 disease.

### C. Training Process

We use ready-made modules of the *PyTorch* library to train the model. Adam was used as an optimization algorithm, and every 100 epochs the learning rate of the classifier decreased by $\gamma = 0.1$. The quality metric was accuracy, i.e., the ratio of correctly predicted classes to the total number of records. The loss function was the binary cross-entropy

\begin{equation*}
{l_n} = - {w_n}\left\{ {\begin{array}{ll}
{\log {x_n}}, &{{y_n} = 1} \\
{\log (1 - {x_n})},&{{y_n} = 0} \end{array}} \right.
\end{equation*}

where ${y_n}$ is the ground truth and ${x_n}$ is the label of predicted class.

The batch size for training the model was 7 records. In total, there were 1000 records of each class in the training set, and this amount of data was achieved by applying augmentations to the original 300 records. Among the augmentations used are adding white noise to the record and obtaining a new spectrogram by averaging two others with the same frequency and time range.

The test sample contained 48 records that were not involved in training the model. In contrast to the learning process, for which the ratio of sick to healthy patient records was artificially changed, in this case 15 records of the COVID category were taken, the rest belonged to the Non-COVID category.

## III. Results

The results of testing the model are demonstrated in Tables I–III. Table I presents the following performance metrics:

\begin{align*}
{\mathop{\rm Accuracy}\nolimits} &= \frac{{TP + TN}}{{TP + FP + TN + FN}},\\
P &= {\mathop{\rm Precision}\nolimits} = \frac{{TP}}{{TP + FP}},\\
R &= {\mathop{\rm Recall}\nolimits} = \frac{{TP}}{{TP + FN}},\\
{{\mathop{\rm F}\nolimits} _1} &= 2 \cdot \frac{{P \cdot R}}{{P + R}},
\end{align*}

where $TP$, $FP$, $TN$, $FN$ denote the number of true and false positives and true and false negatives, respectively.

**TABLE 1.** Quality of the Model

**Accuracy**	**Precision**	**Recall**	**Specificity**	**F_1_**
0.8542	0.7857	0.7333	0.9091	0.7586

A confusion matrix for classification of test sample is reported in Table II. Table III contains information about the performance metrics of models implemented in other projects.

**TABLE 2.** Error Matrix For Proposed RCNN Model

		Predicted classes		
True classes			COVID	Non-COVID
	COVID		11	4
	Non-COVID		3	30

**TABLE 3.** Comparison of the Performance of RCNN Model With Other Projects

Metric	Model				
	Acoustery	Cambridge	AI4COVID-19 Classifiers		
			1	2	3
Accuracy	0.85	-	0.92	0.89	0.93
Precision	0.79	0.63	0.90	0.87	0.91
Recall	0.73	0.84	0.89	0.92	0.95
F_1_	0.76	0.72	0.90	0.89	0.93

## IV. Discussion

The quality of the classification of our system is comparable to the quality achieved in the Cambridge project for the classification of cough into the COVID and Non-COVID categories but is still inferior to the individual classifiers of the *AI4COVID-19* model. However, due to the use of a fixed window in the recurrent network, the quality of the proposed solution does not depend on the operation of the cough detector. In addition, our model excludes the possibility of ambiguity, i.e., in any case, a positive or negative result for COVID-19 will be issued. Another advantage of the proposed RCNN model is that it uses spectrograms of records, while the aforementioned algorithm uses multidimensional matrices of frequency features as input data, which obliges to use Principal Components Analysis to reduce the dimension.

Now, the main problem for diagnosing coronavirus infection using deep learning methods is the lack of training data. First, there is a data imbalance in the collected dataset. Second, records of the Non-COVID class cough often turn out to be unrepresentative due to its artificiality, which reduces the training sample. For these reasons, it is very important to cooperate with medical institutions and doctors. To improve the quality of the model, in addition to the cough records, it is necessary to consider breath records and other patient information.

The results of testing the model led us to the idea of implementing its modified version without the LSTM layer, on which further work on the designing of an application for the differential diagnosis of coronavirus infection will focus. Work should also continue to mark up the collected data to determine representative data and replenish the training sample.

It should be noted that the proposed method for diagnosing COVID-19 is not a substitute for medical testing. However, it can be used to decide whether a patient needs to be tested by PCR. This is especially true for asymptomatic patients. We hope that in the future, equipping doctors with a system registered as medical software will reduce the burden on laboratories, increasing their efficiency. In addition, the proposed system can be used in combination with checking the temperature at the entrance to institutions.

## V. Conclusion

The paper presents a method for differential diagnosis of COVID-19, based on the deep learning techniques. RCNN architecture consists of CNN encoder, LSTM and fully connected layers. The main feature of the proposed model is attention mechanism, the task of which is to determine, based on the significance of a fragment of an audio recording, its contribution to the diagnosis of coronavirus infection. At this stage, the question of the need for the LSTM layer in architecture remains unresolved.

Despite the fact that about 3000 cough records have been collected using the mobile application and open resources, most of them do not belong to the COVID class. Moreover, some records turn out to be unrepresentative, especially for healthy people, whose cough is too feigned. For these reasons, only 300 records were included in the training set, and the ratio of classes had to be artificially changed using augmentations. In the future, we will have to check the quality of the model on a larger test set.

Through collaboration with the hospitals where the data was collected, and the help of doctors in processing the records, it was possible to achieve a model accuracy of 85%. We hope to improve the classification quality of the proposed model after increasing the training set, modifying the architecture, and taking into account all available patient information.
